# Smile enhancement the conservative way: Tooth whitening procedures

**DOI:** 10.4103/0972-0707.58342

**Published:** 2009

**Authors:** Deepika Thosre, Sanjyot Mulay

**Affiliations:** Department of Conservative Dentistry, Endodontics and Aesthetic Dentistry, Dr. D. Y. Patil Dental College and Hospital, Pimpri, Pune - 411 018, India

**Keywords:** 35% carbamide peroxide, non-vital bleaching, in-office power bleaching

## Abstract

This article presents clinical cases in which different bleaching modalities have been used to successfully treat unsightly teeth. Depending upon the type and severity of discoloration, in-office vital and nonvital bleaching procedures were carried out. Discoloration of a single tooth has been managed using nonvital bleaching alone or with a combination of other minimally invasive modalities for an acceptable esthetic outcome. The case selection was done by considering the patient's needs and expectations, the type and cause of discoloration and patient economics. Moreover, prime importance was given to the conservation of the existing tooth structure and acquiring a complete change in the shade of teeth, which was comparable to that of the adjacent teeth. The desire to have a bright smile has become an important esthetic need of patients. The article explores various forms of bleaching and their successful usage in day-to-day clinical practice.

## INTRODUCTION

The desire to have white teeth and thus a more pleasant smile has become an important esthetic need of patients today. Various in-office vital bleaching techniques are effective for teeth with generalized discoloration.[1,2] A single discolored anterior tooth in some patients may stand out and majorly influence the esthetics of smile and thus the confidence of many people. Intra-coronal bleaching of nonvital teeth involves the use of chemical agents within the coronal portion of an endodontically treated tooth to remove tooth discoloration.[3] It may be successfully carried out at various times, even many years after root canal therapy and discoloration. The successful outcome of any of the applied modalities mainly depends on the etiology, diagnosis and proper selection of bleaching materials and the correct clinical technique.[4,5]

## CASE REPORTS

### Case 1

**Figure 1:**
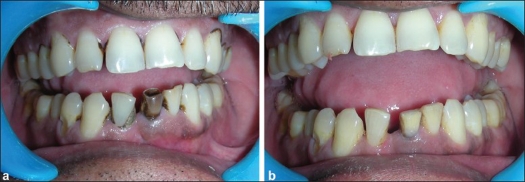
(a) Pre-operative condition, (b) Post-operative result

A 38-year-old male reported to the Department of Conservative Dentistry, Endodontics and Aesthetic Dentistry with a discolored mandibular left central incisor [Figure 1a] and felt conscious due to the unsightly appearance of the tooth and demanded an improvement in his esthetic appearance. The patient presented a history of trauma to the lower anterior region 15 years ago. The treatment protocol followed in this case was root canal treatment followed by walking bleach using 35% hydrogen peroxide.[6]

Patient was familiarized with the possible causes of discoloration, the procedure to be followed, the expected outcome and the possibility of future re-discoloration. Thorough oral prophylaxis was done, and clinical photographs were taken. Root canal treatment was performed for the mandibular left central incisor. In the next appointment, excess Gutta-percha was removed from the access cavity, and it was cleaned. The height of clinical crown was measured with a periodontal probe, and it was made sure that Gutta-percha is removed approximately 2 mm below this level. Light-cured glass ionomer barrier of 2 mm was placed over the Gutta-percha and then cured using an LED light curing unit. Then, 35% hydrogen peroxide gel was placed into the pulp chamber, which was sealed using intermediate restorative material. The patient was followed up after one week.[6]

After three non-vital bleaching sessions, a complete metamorphosis of the discolored tooth was evident and the present color was comparable to that of the adjacent teeth [Figure 1b]. Post-obturation restoration was done using composite resin.[6]

### Case 2

**Figure 2:**
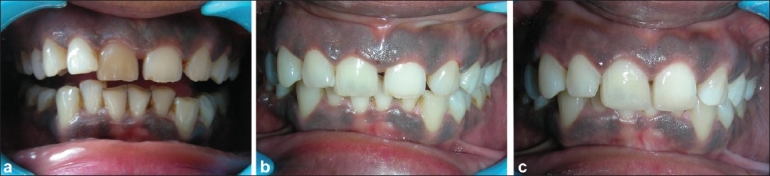
(a) Pre-operative condition, (b) Post bleaching result, (c) Post operative result following gingivectomy

A 28-year-old female reported to the department with a chief complaint of unsightly and short teeth, and she desired an improvement in her esthetic appearance [Figure 2a]. Patient presented a history of trauma to the upper anterior region 4 years ago.[6]

Clinical examination revealed discolored maxillary right central incisor and an asymmetric gingival contour. First, root canal treatment was performed in tooth no. 11. In the next appointment, a lentulo spiral was used to place a light-cured glass ionomer barrier 2 mm below the cemento-enamel junction, and it was shaped like a ski-slope. This was followed by the application of 35% hydrogen peroxide gel in the pulp chamber and sealing it with intermediate restorative material [Figure 2b]. The patient was followed up after a week; the previous bleaching agent was removed, and the chamber was cleaned. A fresh bleaching agent was inserted into the chamber, and the patient was followed up again after a week. The discoloration of the tooth completely disappeared in two sittings. Subsequently, gingivectomy was performed for the maxillary central, lateral and canine of both the sides. This was performed using a BP handle and a no. 12 surgical blade. After 4 weeks of primary gingival healing, post-obturation restoration was done using a composite resin. Further, the midline diastema was minimized [Figure 2c]. The final result was esthetically appealing as requested by the patient, and this was achieved with minimal intervention.[6]

### Case 3

**Figure 3:**
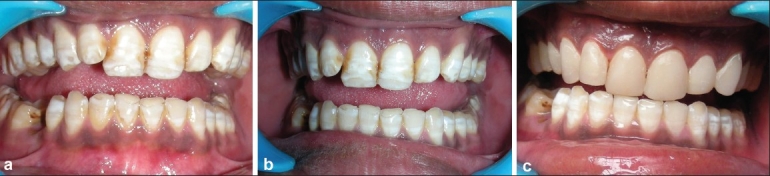
(a) Pre-operative photograph, (b) Post bleaching result, (c) Composite veneers fabricated on maxillary teeth

A 25-year-old male reported to the department with a chief complaint of unsightly teeth due to generalized fluorosis [Figure 3a]. A thorough oral prophylaxis was performed. This was followed by in-office vital bleaching for both the arches to lighten the discoloration. First, a gingival barrier was placed around the anterior teeth along the gingival contour and was light cured. A 35% hydrogen peroxide in-office vital bleaching gel was evenly applied onto the buccal surfaces of teeth [Figure 3b]. This was done for 30 min, and alternate light curing was performed to enhance the bleaching process. All the instructions of the manufacturer were followed. The bleaching agent was then wiped off using a guaze, and patient was asked to rinse. A fluoride gel was then applied evenly onto the bleached teeth. This was followed by composite veneers for the maxillary arch [Figure 3c].[6]

### Case 4

**Figure 4:**
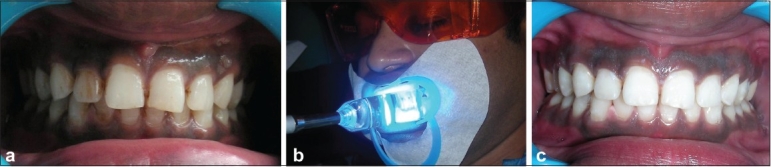
(a) Pre-operative photograph, (b) Application of bleaching agent and activating it using plasma arc light, (c) Post-operative result

A 23-year-old male reported to the department with a chief complaint of yellowish brown discoloration of teeth [Figure 4a] and wanted to have a whiter and brighter smile. After thorough oral prophylaxis, the superficial stains were removed using pumice and a rubber cup. In-office power bleaching was performed for both the arches.[6]

The preoperative shade was assessed using the provided shade guide. A gingival barrier was placed around the teeth of the maxillary arch from the 1^st^ premolar of one side to the that on the other side. This was also done for the mandibular arch. The barrier was light cured. Then, 35% hydrogen peroxide liquid and powder were freshly mixed and applied evenly onto the buccal surfaces of teeth using an applicator brush. Four cycles of power bleaching were carried out using a plasma arc curing light [Figure 4b]. The light was applied 6-7 mm away from the teeth. Strict adherence to the manufacturer's instructions was observed, particularly in relation to the appropriate timing for the materials to remain on the teeth.[6]

The plasma arc light was incident for a period of 10-15 min, and the bleach was removed from the teeth via the high volume aspirator and damp gauze. The teeth were then washed, rinsed and the bleach was reapplied for a further 10 min. The process was repeated for 45 min to 1 hour. The mucosal protectant was removed, and the mouth was rinsed. The teeth were then polished using a diamond polishing paste to achieve an enamel luster [Figure 4c]. The shade of teeth was again assessed and compared preoperatively. A fluoride gel was then applied evenly onto the bleached teeth.[6]

## DISCUSSION

Although a wide arena of esthetic restorative materials are available to us today for the management of discolored anterior teeth, bleaching still remains a viable option in certain cases. A number of factors play an important role in deciding the treatment plan. The patient-related factors for a successful treatment outcome are the patient's needs, age, expectations and affordability. The clinician-related factors include the availability of bleaching materials and a thorough knowledge of the material science, including methodologies and techniques involved.[7,8]

Nonvital bleaching also can be an esthetically pleasing and minimally invasive option for young patients rather than a complete coronal coverage. Intracoronal bleaching of nonvital teeth involves the use of chemical agents within the coronal portion of an endodontically treated tooth to remove tooth discoloration.[9] It may be successfully carried out at various times, even many years after root canal therapy and discoloration. The successful outcome depends mainly on the etiology, correct diagnosis and proper selection of bleaching technique. Walking bleach is preferred as it requires less chairtime and is safer and more comfortable for the patient.[10]

The indications of nonvital bleaching include the following:

- Discoloration of pulp chamber origin.[1]
- Dentin discolorations.[1]
- Discolorations not amenable to extracoronal bleaching.[1]

The contra-indications of nonvital bleaching include the following:

- Superficial enamel discoloration.[1]
- Defective enamel formation.[1]
- Severe dentin loss.[1]
- Presence of caries.[1]
- Discolored composites.[1]

In-office bleaching is the most commonly used method; in particular, in-office power bleaching is seen to impart tremendous patient satisfaction. In-office bleaching is useful for the removal of stains throughout the arch or even treating specific areas of a single tooth (such as in some types of fluorosis). The dentist is in complete control of the process throughout treatment.[11] This provides the advantage of continuing the treatment or terminating the bleaching process at any time. In-office bleaching is usually a fast process that the results are evident even after a single visit. Many patients prefer bleaching by the dental professional because it requires less active participation on their part.[12]

The indications of in-office bleaching include the following:

- Developmental or acquired stains.[1]
- Stains in enamel and dentin.[1]
- For removing yellow brown stains.[1]
- Yellowing of teeth due to aging.[1]
- For blending white color changes.[1]
- Mild to moderate tetracycline changes.[3]

The contra-indications of in-office bleaching include the following:

- Tetracycline staining.[1]
- Pitting hypoplasia.[1]
- Teeth with deep and surface cracks and fracture lines.[1]
- Teeth with large anterior restorations.[1]
- Periapical pathology.[1]
- Teeth exhibiting extreme sensitivity to heat, cold, touch and sweetness.[1]
- Patients who smoke.[1]
- Patients with unrealistic expectations about the anticipated esthetic result.[3]
- Teeth with excessive tooth surface loss due to attrition, abrasion and erosion.[3]

Certain side effects and problems associated with bleaching should always be kept in mind whilst performing the procedure.[6]

### Gingival and soft tissue irritation

Strong concentrations of 35% hydrogen peroxide can cause soft tissue damage, gingival ulceration and skin burns. Normally these burns appear as a white lesion in the area, followed by a red rim. These disappear after a few minutes, heal quickly and do not cause any permanent damage. If such lesions occur, the patient should be told, shown and reassured. Therefore, a gingival barrier is mandatory.[1]

### Altered taste/sensation

Some patients report a metallic taste sensation immediately after bleaching; however, this normally disappears after few hours.[1]

### Tooth sensitivity

- If this has occurred, the patient should be reassured that this is a common side effect and will disappear after bleaching.[1]
- Patients should be reassured that the side effects are minor and transient and will disappear after the completion of treatment.[1]

Moreover, depending upon the clinical condition, a synergistic approach of combining bleaching with other modalities such as micro-abrasion and composite veneers can help in gaining an excellent clinical outcome. Taking into account the increasing esthetic demand of the patients, this approach proves to be conservative and simple for the successful management of unsightly teeth.[6]

## References

[1] Greenwall L (2001). Bleaching techniques in restorative dentistry.

[2] Sturdevant CM, Roberson TM, Heymann HO, Sturdevant JR The art and science of operative dentistry.

[3] Feinman RA, Goldstein RE, Garber DA (1987). Bleaching teeth. Quintessence Int.

[4] Watts A, Addy M (2001). Tooth discolouration and staining: A review of the literature. Br Dent J.

[5] Christensen GJ (1997). Bleaching teeth: Practitioner trends. J Am Dent Assoc.

[6] Goldstein, Garber (1995). Complete dental bleaching.

[7] Pearson H (1958). Bleaching of discolored pulpless tooth. J Am Dent Assoc.

[8] Baratieri LN, Ritter AV, Monteiro S, Andrada MA, Vieria LC (1995). Non vital tooth bleaching: Guidelines for the clinician. Quintessence Int.

[9] Sulieman M, Addy M, Macdonald E, Rees JS (2005). The bleaching depth of a 35% hydrogen peroxide based in-office product: A study *in vitro*. J Dent.

[10] Chng HK, Ramli HN, Yap AU, Lim CT (2005). Effect of hydrogen peroxide on intertubular dentine. J Dent.

[11] Goldstein CE, Goldstein RE, Feinman RA, Garber DA (1989). Bleaching vital teeth: State of the art. Quintessence Int.

[12] Suleiman M, Addy M, Macdonald E, Rees JS (2004). A safety study *in vitro* for the effects of an in-office bleaching system on the integrity of enamel and dentine. J Dent.

